# Increased toxin expression in a *Clostridium difficile mfd* mutant

**DOI:** 10.1186/s12866-015-0611-5

**Published:** 2015-12-18

**Authors:** Stephanie E. Willing, Emma J. Richards, Lluis Sempere, Aaron G. Dale, Simon M. Cutting, Neil F. Fairweather

**Affiliations:** Department of Life Sciences, Centre for Molecular Bacteriology and Infection, Imperial College London, London, SW7 2AZ UK; School of Biological Sciences, Royal Holloway University of London, Egham, Surrey TW20 0EX UK

**Keywords:** *Clostridium difficile*, Toxin A, Toxin B, Transcriptional roadblock, *mfd*, CcpA, Transcription-repair coupling factor

## Abstract

**Background:**

The symptoms of *Clostridium difficile* infection are mediated primarily by two toxins, TcdA and TcdB, the expression of which is governed by a multitude of factors including nutrient availability, growth phase and cell stress. Several global regulators have been implicated in the regulation of toxin expression, such as CcpA and CodY.

**Results:**

During attempts to insertionally inactivate a putative secondary cell wall polysaccharide synthesis gene, we obtained several mutants containing off-target insertions. One mutant displayed an unusual branched colony morphology and was investigated further. Marker recovery revealed an insertion in *mfd,* a gene encoding a transcription-coupled repair factor. The *mfd* mutant exhibited pleiotropic effects, in particular increased expression of both toxin A and B (TcdA and TcdB) compared to the parental strain. Western blotting and cellular cytotoxicity assays revealed increased expression across all time points over a 24 h period, with inactivation of *mfd* resulting in at least a 10 fold increase in cell cytotoxicity. qRT-PCR demonstrated the upregulation of both toxins occurred on a transcriptional level. All effects of the *mfd* mutation were complemented by a plasmid-encoded copy of *mfd*, showing the effects are not due to polar effects of the intron insertion or to second site mutations.

**Conclusions:**

This study adds Mfd to the repertoire of factors involved in regulation of toxin expression in *Clostridium difficile*. Mfd is known to remove RNA polymerase molecules from transcriptional sites where it has stalled due to repressor action, preventing transcriptional read through. The consistently high levels of toxin in the *C. difficile mfd* mutant indicate this process is inefficient leading to transcriptional de-repression.

## Background

*Clostridium difficile* is an anaerobic, spore-forming Gram-positive pathogen that is now recognized as the leading cause of antibiotic-associated diarrhea in health care settings [1]. The incidence and apparent severity of *C. difficile* infection (CDI) rose in the mid-2000s, in part due to the circulation of strains resistant to the newer fluoroquinolone antibiotics [2, 3]. The infectious agent is the spore [4], which is remarkably resistant to heat, disinfectants and antimicrobial agents. Treatment of patients with antibiotics dramatically alters their gut microbiota [5] and this perturbation can cause loss of colonization resistance, allowing indigenous and exogenous pathogens to colonize and cause disease [6]. Under these conditions, spores of *C. difficile* can germinate in the gut, and the resulting vegetative cells proliferate in high numbers. Vegetative cells and spores are excreted in large numbers and subsequent spore transmission can cause localized epidemics in health care settings.

The major virulence factors are two large toxins, TcdA (toxin A) and TcdB (toxin B). All toxigenic strains produce toxin B and a large percentage of strains also produce toxin A [7]. These toxins have a similar structure and mode of action; the toxins are large, multi-domain proteins encoding glucoslytransferase and cysteine protease activities together with a repetitive receptor binding domain [8]. The toxins are released from bacteria in the gut lumen and are taken up by receptor-mediated endocytosis into enteric cells. Recent evidence suggests that additional receptor binding activity could be encoded within the central translocation domain [9]. Once internalized into vesicles, the cysteine protease activity is required to release the N-terminal glucosyltransferase domain from the adjacent protease domain, an activity dependent on cytosolic inositol-6-phosphate [10].

The toxin genes *tcdA* and *tcdB* are encoded within a genomic locus, PaLoc, with three other genes: *tcdR, tcdE* and *tcdC* [11]. The regulation of toxin synthesis is complex, with multiple forms of regulation evident. Toxin expression is related to the growth phase of the bacterium, with maximal expression occurring in the late-logarithmic phase of growth [12]; a quorum sensing molecule that may be the main mediator of this level of regulation was recently identified [13]. The toxins are under the control of the Gram-positive global transcriptional regulator CodY [14, 15]. CodY binds to the promoter upstream of *tcdR* [15], a gene specifying an alternative sigma factor necessary for transcription from the *tcdA* and *tcdB* promoters, as well as to the *tcdR* promoter itself [16]. CodY also regulates over 150 other genes in *C. difficile*, and likely functions to monitor the expression of genes in response to nutrient sufficiency [14]. Several environmental and nutritional factors influence toxin expression including sub-inhibitory levels of antibiotics, the redox potential and the amino acid content of the medium. Spo0A, a transcriptional regulator essential for sporulation in *B. subtilis* and *C. difficile*, negatively regulates toxin production, with *spo0A* mutants producing increased levels of toxins A and B in some strains of *C. difficile* [4, 17]. Notably, toxins A and B are also subject to carbon catabolite repression (CCR), their expression being markedly reduced in the presence of rapidly metabolizable sugars such as glucose [12]. In Gram-positive bacteria CCR is mediated by the transcriptional regulator CcpA. Genetic inactivation of components of the CCR signal transduction pathway results in de-repression of toxin expression in the presence of glucose, showing uptake of glucose is required for toxin repression [18]. CcpA binds to genetic elements within the *C. difficile* PaLoc, but recognizes sequences distinct from those of the well characterized *cre* (*c*atabolic *r*epression *e*lements) sites recognized by *B. subtilis* CcpA. In *C. difficile*, five *creCD* binding sites are found within the PaLoc: two upstream of the translational start site of *tcdR*, one upstream of *tcdB*, one inside *tcdA* and one inside *tcdC* [18]. Finally, *Clostridium difficile* toxins are regulated by the second messenger cyclic di-GMP [19]. Increased intracellular levels of cyclic di-GMP repress expression of *tcdA, tcdB* and *tcdR,* an effect likely mediated by the alternative sigma factor SigD. SigD directly activates *tcdR* expression and, as di-GMP lowers the levels of SigD, TcdR levels fall and toxin gene expression is reduced.

Transcription-repair coupling factor (TRCF), also known as Mfd in bacteria (*m*utation *f*requency *d*ecline), is a highly conserved protein that links the processes of nucleotide excision repair and transcription elongation [20]. In *E. coli* the Mfd protein is 130 kDa and consists of 8 domains with two functional modules; an N-terminal module homologous to UvrB that recruits DNA repair proteins and a C-terminal module that possesses ATPase-dependent DNA translocase motor activity [21, 22]. RNA polymerase (RNAP) molecules that stall at DNA lesions are removed by the translocase activity of Mfd. The removal of stalled RNAP is not confined to DNA lesions, but is applicable to RNAP stalled for other reasons including nucleotide starvation or by blockage by other DNA bound proteins such as transcriptional repressors (reviewed in [23]). In *B. subtilis*, a Tn10 insertional mutation within *mfd* has been shown to partially relieve CCR at *cre* elements that are sufficiently promoter distal to form transcriptional roadblocks by recruitment of CcpA [24]. Similarly, Mfd inactivation in *B. subtilis* partially relieves CodY-mediated transcriptional repression at post-promoter sites where this regulator can form transcriptional roadblocks that prevent RNA polymerase progression [25].

In this study, we characterize an insertional mutant in the *C. difficile mfd* gene. This mutant has abnormal colony morphology and produces a higher level of toxins A and B than its parent strain.

## Results

**Fig. 1:**
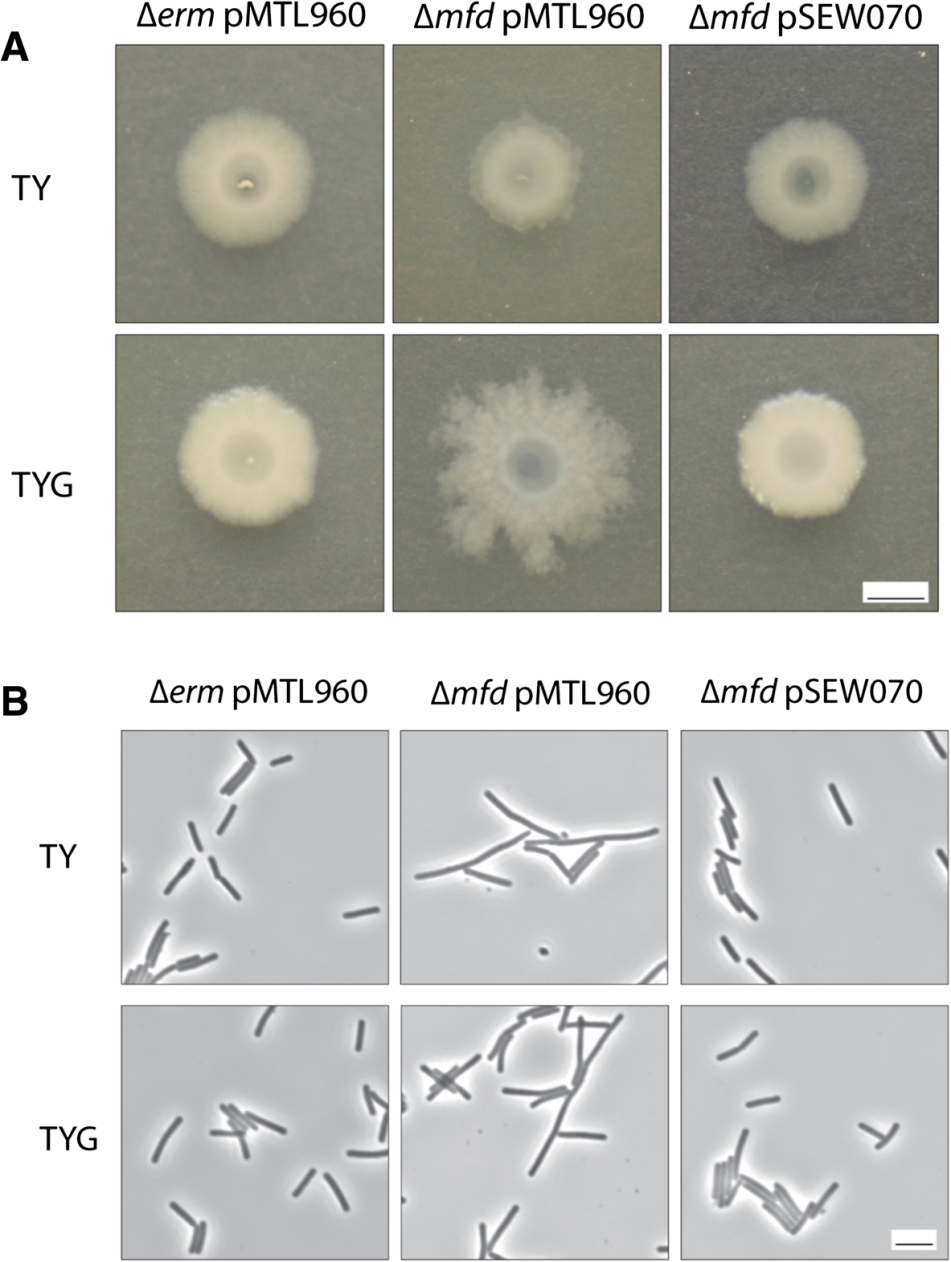
Colony and cellular morphology of the *C. difficile mfd* mutant. **a** Liquid cultures (1 μl) of strains 630Δe*rm* and the *mfd* mutant containing plasmid vector alone (pMTL960) or with the *mfd* gene (pSEW070) as appropriate were spotted on to TY or TYG agar and grown for 3 days. Representative colonies were photographed. Bar = 1 cm. **b** Cultures of parental strain 630Δe*rm* and the *mfd* mutant containing pMTL960 vector or pSEW070 were grown in TY or TYG broth and photographed under phase contrast microscopy. Bar = 5 μm

As part of a study into the *C. difficile* cell wall, we attempted to use a group II intron and an erythromycin resistance marker to target and inactivate the gene CD2775, located in a gene cluster downstream of *slpA* encoding the major S-layer protein in strain 630Δ*erm* [26]. No insertions into CD2775 were obtained, perhaps because this gene is essential under the conditions of growth we employed. However some erythromycin resistant mutants were obtained, presumably where the group II intron had inserted into genes containing sequences related to the target sequence of CD2775. One of these mutants, designated 630–911 and subsequently *mfd::erm* (see below), had an interesting colony morphology on BHIS agar, with colonies displaying a highly branched structure in comparison to the compact colonies obtained with the wild type parent 630Δ*erm* (data not shown). Colony morphologies were studied further using media of a more defined composition. On TY agar (without glucose), the *mfd* mutant colonies appeared slightly smaller than the parental strain, but on TYG agar (containing glucose) the mutant colonies were considerably larger and displayed a highly branched structure (Fig. 1a). Cells from cultures grown in TY or TYG liquid cultures were examined using phase contrast microcopy. When grown in either medium, the cells of the mutant appeared longer than the parental strain, up to 10 μm in length and slightly more curved (Fig. 1b).

### Strain 630–911 contains a defect in the *mfd* gene

**Fig. 2:**
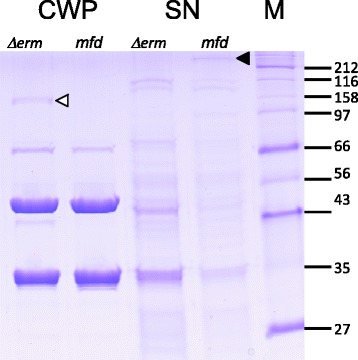
Cell wall proteins and culture supernatants of the *mfd* mutant. Cultures were grown in BHIS broth and cell wall proteins and culture supernatants were prepared and analyzed by 12 % SDS-PAGE. A high molecular weight band (▶) was observed in the culture supernatant of the *mfd* mutant, which was identified as toxin A. The phase variable CwpV protein is observed in the parental Δ*erm* culture (▷). CWP, cell wall proteins; SN, culture supernatants; M, molecular weight markers

To investigate the phenotype of the mutant further, cell wall extracts and supernatants were prepared from cultures of the 630–911 *(mfd)* mutant and the parental strain grown in BHIS broth and were analyzed by SDS-PAGE (Fig. 2). No differences in the cell wall protein profiles were apparent, aside from the appearance of bands at ~42 kDa and ~116 kDa in the Δ*erm* strain. These are the products of *cwpV*, a phase variable gene that results in variable levels of CwpV proteins in the cell wall and which undergoes intramolecular autoproteolysis [27, 28]. However, in the supernatants of the *mfd* mutant, an increased level of a large protein of over 212 kDa was observed, which was not seen in strain 630Δ*erm*. Mass spectrometry of a tryptic digest of this protein revealed it to be derived from toxin A (TcdA; data not shown and see below).

To localize the mutation in the mutant, genomic DNA was prepared and digested with EcoRV, ClaI or HindIII. The digests were ligated with plasmid pBluescript cleaved with the same enzyme and, after ligation, *E. coli* transformants were selected on erythromycin to clone the locus into which the intron and the *erm* gene had inserted. Sequence analysis of plasmids revealed the presence of the *erm* gene and *C. difficile* DNA encoding the ORF CD3501, indicating this gene as the site where the intron had inserted. Insertion within CD3501 between basepairs 1412 and 1413 was subsequently confirmed by PCR using primers flanking the insertion site, and Southern blot confirmed this to be the only ClosTron insertion site (data not shown). CD3501 encodes *mfd*, a transcription-repair coupling factor (TCRF) [29]. TCRFs are widely conserved in nature and function to relieve stalled RNA polymerase molecules at regions of DNA damage, increasing the rate of excision of UvrABC exonucleases (see Introduction).

**Fig. 3:**
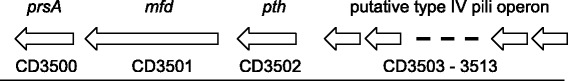
Genome region of *C. difficile* 630 at the *mfd* locus. Downstream of *mfd* (CD3501) is *prsA* (CD3500) a putative chaperone, while upstream is *pth* (peptidyl-tRNA hydrolase; CD 3502) and a multi-gene type IV pili locus (CD3503 – CD3513). All genes are transcribed in the same direction (right to left in this diagram). CD gene numbers are given below the genetic symbols

The *mfd* gene is present in a region of the chromosome containing a putative type IV pilus operon, a peptidyl tRNA isomerase *pth* and the putative chaperone *prsA* (Fig. 3). To ensure that the mutant phenotype observed was not due to a polar effect on a downstream gene, the *mfd* gene was complemented by cloning the wild-type *mfd* gene from strain 630Δ*erm* in an *E. coli – C. difficile* shuttle vector under control of the constitutive promoter P*cwp2* [30] to create pSEW070. When introduced into the *mfd* mutant, pSEW070 conferred a wild-type colony phenotype (Fig. 1a) and under phase contrast microscopy the cells had the appearance of the wild type strain, with no elongated cells visible (Fig. 2b). When culture supernatants were analyzed by SDS-PAGE, the >212 kDa TcdA band was no longer visible in the complemented strain (see below) demonstrating that these phenotypes are due solely to the disruption of *mfd.* Western blotting with anti-TcdA and anti-TcdB confirmed the over-expression of TcdA and demonstrated that TcdB was also over-expressed (see below).

### The *mfd* mutant over-produces toxins TcdA and TcdB

**Fig. 4:**
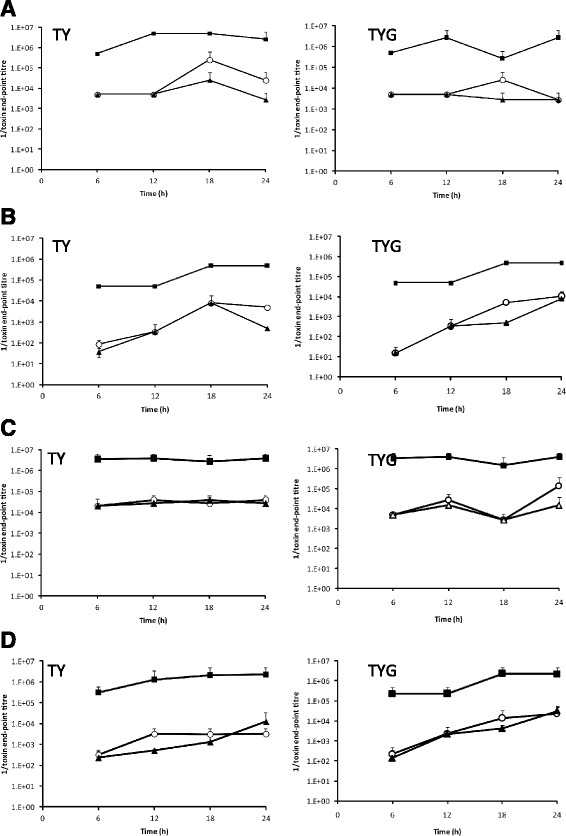
Cytotoxicity levels of toxins A and B in the *mfd* mutant. Cultures of 630Δ*erm,* the *mfd* mutant or the complement were grown in TY or TYG media. Cell lysates and culture supernatants were prepared at various time points. Samples were normalized for optical density of the culture and analyzed for the presence of toxins A and B by cytotoxicity on HT29 cells (toxin A) or Vero cells (toxin B). Panels: **a** bacterial lysates on Vero cells; **b** bacterial supernatants on Vero cells; **c** bacterial lysates on HT29 cells; **d** bacterial supernatants on HT29 cells. Samples were standardized by OD at point of collection. ○, 630Δ*erm;* ■ *mfd* mutant; ▲ complement. Data represents the mean of two biological repeats and at least two technical repeats and error bars represent standard deviations

The quantities of functionally active TcdA and TcdB present in bacterial cell lysates and in culture supernatants were determined by a cytotoxicity assay using HT29 and Vero cells, lines that are differentially sensitive to TcdA and TcdB, respectively [31, 32]. When applied to these cell lines, the toxins induce cell rounding which can be scored by enumeration of cells by microscopy. As shown in Fig. 4, the levels of TcdA and TcdB in the *mfd* mutant were consistently higher than in the parental strain 630Δ*erm* at all time points. The toxin levels observed in the *mfd* mutant varied according to time point and culture conditions, but in all cases were over 10 fold higher than the wild-type or genetically complemented samples. Although variations in the fold increase of cytotoxicity of the *mfd* mutant to the parental control and the complemented mutant were evident, the *mfd* mutant consistently produced higher levels of TcdA and TcdB throughout growth, and this was seen in both cell lysates and in culture supernatants. Importantly, in the complemented mutant the levels of both toxins were similar to the parental cells.

**Fig. 5:**
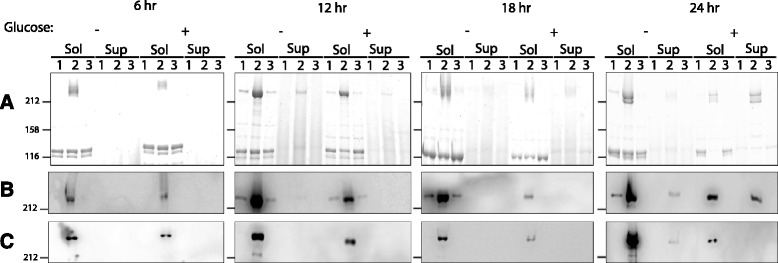
Toxin A and B production in the *mfd* mutant. Cultures of 630Δ*erm* (1) the *mfd* mutant (2) and the complemented strain (3) were grown in TY or TYG media. Cell lysates and culture supernatants were prepared at various time points and analyzed by Coomassie Blue stained 6 % SDS-PAGE (**a**) and by Western blotting using anti-toxin A (**b**) or anti-toxin B antibodies (**c**). The addition of glucose to the TY media to form TYG is indicated. The stained bands migrating above the 212 kDa marker in panel **a** are presumed to be a mixture of toxin A and toxin B, as these proteins tend to co-migrate and smear on gels

To further investigate the production of toxins TcdA and TcdB by the *mfd* mutant, 630Δ*erm*, the *mfd* mutant and its complement containing pSEW070 were grown in TY or TYG and toxin levels in cell lysates and in culture supernatants analyzed by Western blotting. As shown in Fig. 5a, a prominent band over 212 kDa was visible by Coomassie blue staining of cell lysates of the *mfd* mutant extracted at all time points. This band was identified as TcdA (see Fig. 2). The band was not visible in the parental strain or the complemented mutant at 6 h, but was visible in the wild-type strain and complemented mutant at 12, 18 and 24 h, but at considerably lower intensity than in the *mfd* mutant strain. In the culture supernatants, an equivalent band was visible at 12, 18 and 24 h, but again at all time points the intensities of the bands seen from the *mfd* mutant were greater than those in the wild-type or complemented strain. As TcdA and TcdB are known to be co-regulated [12] and to investigate whether TcdB expression was also up-regulated in the *mfd* mutant, we used Western blotting to analyse the expression of both toxins. The results were similar to those seen by Coomassie blue staining and showed high level expression of TcdA in the *mfd* mutant compared to the wild type or complemented strains (Fig. 5b). TcdB was also detectable by Western blotting (Fig. 5c). Similarly to TcdA, the levels of TcdB were maximal in the cell lysates of the *mfd* mutant compared to the parental or complemented controls. Levels of expression did not completely mirror those of TcdA, as levels were maximal at 24 h and those at 18 h appeared lower than at 12 h. Only at 24 h was expression of TcdB apparent in the supernatants, and only in the *mfd* mutant strains.

**Fig. 6:**
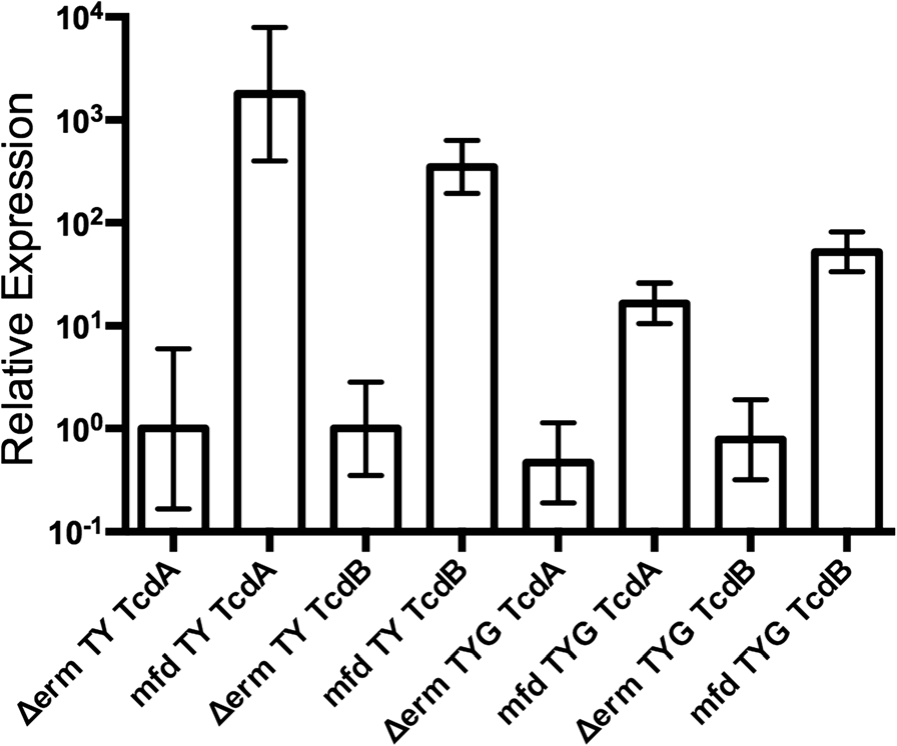
Transcription of tcdA and tcdB in the *mfd* mutant. The rates of transcription of *tcdA* and *tcdB* were measured by quantitative RT-PCR in the wild type (Δ*erm*) and the *mfd* mutant strain grown in TY and TYG media. Standard error of the mean error bars are indicated

To verify that the increased production of toxins A and B was due to increased transcription, RNA was extracted from 630*∆erm* and the *mfd* mutant after 6 h growth in either TY or TYG, and quantitative RT-PCR performed. As shown in Fig. 6 a significant increase in transcript levels of both *tcdA* and *tcdB* was seen in the *mfd* mutant compared to 630Δ*erm* strain when grown in TY (*p* ≤0.01) or TYG (*p* < 0.05). This demonstrates that an increase in toxin production is occurring on a transcriptional level in the *mfd* mutant both in the presence and absence of glucose. Overall, our results demonstrate increased expression of toxins TcdA and TcdB in the *mfd* mutant, and that this is due solely to the presence of the insertional mutation in the *mfd* gene.

### The *mfd* mutant does not exhibit a pleiotropic phenotype

**Fig. 7:**
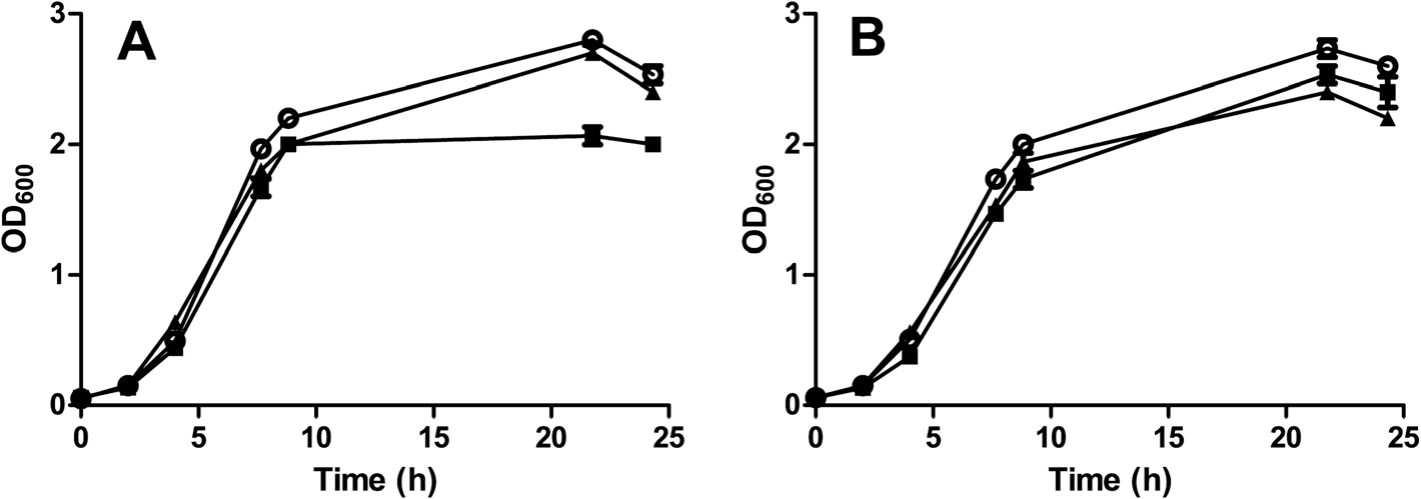
The *mfd* mutant exhibits a growth defect in rich medium. Cultures of 630Δ*erm* (○), the *mfd* mutant (■) and the complemented strain (▲) were cultured in TY (**a**) or TYG (**b**) media containing 15 μg/ml thiamphenicol and their growth measured. The 630Δ*erm* and *mfd* strains contained the plasmid pMTL960 and the complemented strain the plasmid pSEW070

As TCRFs can be involved in a diversity of cellular activities [24, 25] we investigated whether our *C. difficile mfd* mutant exhibited phenotypes other than the unusual colony morphology and toxin upregulation. The growth rates of the wild–type and mutant strains were similar, however when grown in TY medium (Fig. 7a), the *mfd* mutant ceased growth almost completely at the end of exponential phase (at 10 h) whereas the wild type strain and the complemented strain continued to grow for a further 12 h (Fig. 7b). In *B.* s*ubtilis*, it has been reported that inactivation of *mfd* results in a 40 % decrease in sporulation efficiency compared to WT [24]. However, in contrast to *B. subtilis*, the *C. difficile mfd* mutant did not show a defect in sporulation (data not shown). Finally, we also investigated flagella-mediated motility, and found no obvious defect in the *mfd* mutant (data not shown).

## Discussion

Our use of marker recovery revealed that an uncharacterized mutation was within *mfd* (CD3501), encoding a TCRF that directs the preferential repair of template strand DNA of transcribed genes following certain types of DNA damage. In other species, it is known that Mfd acts through the combined functions of a C-terminal domain which can interact with, and subsequently displace, stalled RNA polymerases and an N-terminal domain with homology to UvrB which recruits the nucleotide excision repair machinery, UvrABC, via interaction with UvrA [33]. Thus, with the combined actions of these two domains, Mfd functions to restore transcription and repair mutations before they become fixed in the genome by replication. While the role of Mfd as a repair factor is well established, it has also been shown to have a role in adaptive mutagenesis. In *B. subtilis* the Mfd protein is important for stationary phase mutagenesis [34] and in *Campylobacter jejuni* Mfd promotes fluroquinone resistance generating mutations [35].

In *Bacillus subtilis,* a forward genetics screen for mutants deficient in CCR isolated an *mfd* mutant [24]. In this mutant, partial relief of CCR occurred for genes with *cre* sites downstream of the promoter, but not those with promoter proximal *cre* sites. This suggested that these *cre* sites lead to RNA polymerase stalling at a roadblock, which can be relieved by Mfd. Without Mfd to release the polymerase, the RNA polymerase will in some instances eventually overcome the roadblock as the repressor protein dissociates, resulting in increased transcription of the gene [35]. Promoter proximal sites prevent transcription initiation and therefore do not cause RNA polymerase to stall, requiring no involvement from Mfd. Similarly, Mfd has been implicated in CodY-mediated repression of genes containing downstream CodY-binding sites [25]. The ability of the Mfd to displace RNA polymerases stalled at transcriptional roadblocks makes Mfd important in maintaining the efficacy of this type of transcriptional repressor.

How does Mfd act in controlling the level of toxin gene expression in *C. difficile*? CodY and CcpA are known repressors of toxin production and binding sites within the PaLoc have been identified for both these proteins. It is possible that Mfd acts by relieving RNA polymerase molecules stalled at roadblocks created by CodY and CcpA. This is consistent with the observed increases in transcription, and the large increases in toxin A and toxin B production in the *mfd* mutant. In our experiments we saw only a small effect of glucose in repression of toxin production in the wild-type cultures, which complicates the analysis of the *mfd* mutant in regard to the role of CcpA. In the *C. difficile mfd* mutant it is apparent that glucose-independent de-repression occurs, as we observe increased transcription of *tcdA* and *tcdB* and increased production of both toxins as measured by cell cytoxicity and Western blotting in the *mfd* mutant compared to the wild type when cultures are grown in TY medium. A similar observation was made for a *ccpA* mutant and it was suggested that CcpA could also act in a glucose-independent manner [36]. It is possible we are witnessing a similar effect here. Alternatively, it is possible that relief of CodY or other as yet unknown transcription roadblocks is also occurring [15].

We have not been able to identify the factor responsible for the colony morphology phenotype or the elongated cells. However, relief of transcriptional roadblocks is certain to have impacts on genomic loci other than the PaLoc. In contrast to *B. subtilis,* we did not observe a defect in sporulation for the *mfd* mutant, adding to the many differences observed in the sporulation pathway between *C. difficile* and *B. subtilis* [37]. Importantly, the observed phenotypes of the *mfd* mutant were complemented upon introduction of a plasmid-encoded copy of *mfd.* This study adds another layer to the regulatory network governing toxin synthesis and secretion and is to our knowledge the first direct implication of Mfd with toxin production and hence virulence of this pathogen. Finally we note that an *mfd* mutant could be of utility in producing increased levels of toxins for commercial applications.

## Conclusions

We have identified that a mutation in the *C. difficile mfd* gene results in abnormal colony morphology and a large increase in the production of toxins A and B. Increased transcription of the *tcdA* and *tcdB* genes is observed, suggesting that in the *mfd* mutant there is relief of transcriptional repression perhaps mediated by CcpA or CodY.

## Methods

### Bacterial strains, plasmids and culture conditions

*C. difficile* strains and plasmids are described in Table 1. Strains were grown and maintained at 37 °C in a Whitley DG250 anaerobic workstation under anaerobic conditions (10 % H_2_, 10 % CO_2_, 80 % N_2_) (D. Whitley, Yorkshire, UK). *C. difficile* strains were cultured in tryptose-yeast (TY) medium (3 % Bacto tryptose, 2 % yeast extract), TYG (TY supplemented with 0.5 % filter sterilized glucose) or BHIS (3.7 % brain heart infusion, 0.5 % yeast extract and 0.1 % cysteine). For visualization of *C. difficile* colony morphology 3 μl of each strain, normalized to OD 0.3, was spotted on to TY or TYG agar, grown for 3 days and imaged using a Canon EOS 450D camera. For solid media, agar was added to a final concentration of 1.5 %. For plasmid maintenance, media were supplemented with 15 μg/ml thiamphenicol when necessary. *E. coli* strains were grown in L-broth or on L-agar as described [38] and plasmids maintained by chloramphenicol (30 μg/ml). For visualization of *C. difficile* cell morphology, strains were grown overnight in either TY or TYG and imaged using a Nikon Eclipse E600 microscope fitted with a Nikon DMX1200 camera.

**Table 1:** Strains and plasmids used in this study

Strain or plasmid	Relevant characteristics	Source or reference
*C. difficile* strains		
630Δ*erm*	Wild-type strain	Obtained from Peter Mullany [29]
630-911 (*mfd* mutant)	630Δ*erm mfd::ermB*	This study
630 *tcdA tcdB*	630Δ*erm tcdA*::*catP tcdB*::*erm*	Obtained from Nigel Minton; [43]
Plasmids		
pMTL960	Plasmid vector for complementation studies	Obtained from Nigel Minton; [38]
pSEW070	pMLT960 carrying wild type *mfd* gene under control of Pc*wp2*	This study

### Genetic techniques

*C. difficile* genomic DNA was isolated as described previously [39] and was cleaved and ligated using conventional procedures [40]. To obtain fragments for cloning, PCR reactions were performed using KOD Hot Start polymerase (Novagen) using primers as detailed below. Standard PCR was performed using Taq polymerase (Sigma). For marker recovery, *C. difficile* 630Δ*erm mfd::erm* genomic DNA was digested with restriction endonucleases and the fragments cloned into pBluescript cleaved with the same enzymes. After ligation, the products were transformed into *E. coli* NovaBlue (Merck) and transformants selected on L-agar containing 500 μg/ml erythromycin. Plasmid pSEW070 was created by amplification of the *mfd* gene from *C. difficile* 630Δ*erm* using KOD polymerase with primers NF2234 and NF2298 and cloning into pMTL960 using the BamHI and SacI sites. Plasmids were conjugated into *C. difficile* firstly by transforming into *E. coli* CA434, selecting for chloramphenicol (30 μg/ml) resistance followed by conjugation into *C. difficile* 630Δ*erm* and selection for thiamphenicol resistance (15 μg/ml) in the presence of clycloserine (250 μg/ml) to kill the *E. coli* donor cells. ClosTron mutants were created using the methods described [41, 42]. Briefly, the L1.LtrB intron present in plasmid pMTL007C-E5 was retargeted to CD2775 using the ClosTron web site (www.clostron.com) and the resulting plasmid pSEW035 constructed by DNA2.0. pSEW035 was transformed into *E. coli* CA434 and then conjugated into *C. difficile* 630Δ*erm* selecting for thiamphenicol resistance (15 μg/ml). Thiamphenicol resistant colonies were re-streaked on to erythromycin (5 μg/ml) to obtain colonies containing chromosomally integrated introns.

### RNA isolation

RNA for quantitative real time PCR was extracted from 630 Δ*erm* and the *mfd* mutant following 6 h growth in TY or TYG. 5 ml of culture was added to 10 ml of RNA protect (Qiagen) in the anaerobic chamber before 10 min centrifugation at 5000 x g, 4 °C. RNA was purified using the FastRNA Pro Blue kit (BIO 101 Systems) and a FastPrep-24 automated homogenizer (MP Biomedical, 45 m/s, for 3  cycles), followed by DNase treatment (TURBO DNA-free, Applied Biosystems). To verify removal of DNA, 16 S rRNA PCR amplification was carried out with 1 μg of RNA. 1 μg of RNA was processed using RETROscript First Strand Synthesis Kit (Ambion).

### Quantitative RT-PCR

Quantitative real time PCR was performed using SYBR Green JumpStart Taq Ready Mix (Sigma), 50 mM of gene specific primers (Table 2), 2 μl of cDNA obtained from 1 μg of RNA as described above, and a Rotor-Gene 6000 real -time rotary analyzer (Corbett). SYBR® Green reaction mixtures contained 500 nM of each primer in a final volume of 20 μL and were incubated for ten minutes at 95 °C, 40 cycles at 95 °C for 30 s, 48 °C for 30 s, 72 °C for 60 s, and a final extension step at 72 °C for 3 min. Melting curves were performed from 50 to 99 °C reading fluorescence at 0.5 °C intervals. Mean Ct values were normalized to *rpoB*, which was amplified using primers SW82 and SW83. Each reaction was performed in technical duplicate and relative expression values reported are representative of two biological replicates.

**Table 2:** Primers used in this study

Primer name	Sequence (5’ to 3’)	Used for
SW78	TCTACCACTGAAGCATTAC	qPCR for *tcdA*
SW79	TAGGTACTGTAGGTTTATTG	qPCR for *tcdA*
SW80	ACCATATAGCTTTGTGATAGTGAAGGAAA	qPCR for *tcdB*
SW81	AAGAACTACATCAGGTAATTCAGATACAAA	qPCR for *tcdB*
SW82	GGATGATATGATGAAGGTTAGAAACCT	qPCR for *rpoB*
SW83	CCCAATCCAAGTTCTTCTAGTTTTTG	qPCR for *rpoB*
NF408	TCTTGAATATCAAAGGTGAGCCAGTACA	16S RNA amplfication
NF409	TACAGCGTGGACTACCAGGGTATCTAAT	16S RNA amplfication
NF2234	GATCGAGCTC AATATAATGGATAGTGAGAG	*mfd* amplication
NF2298	GATCGAGCTC AATATAATGGATAGTGAGAG	*mfd* amplication

### Western blotting and mass spectrometry

SDS-PAGE was carried out as described previously [38]. 6 % or 12 % acrylamide was used in the resolving gel as specified in the figure legends. For immunoblot analysis, proteins were transferred to Immobilon-PVDF membranes (Millipore) using a three-buffer semi-dry method according to the instructions provided by the manufacturer. Anti-Toxin A (PCG4.1, Novus Biologicals) was used in a 1:1000 dilution and rabbit anti-Toxin B (a gift from Ingo Just, Hannover Medical School) in a 1:5000 dilution in 3 % milk powder (VWR) in PBS (VWR). Primary antibodies were detected by using horseradish peroxidase (HRP)-conjugated goat anti-mouse antibody at 1:1000 (Dako) and the SuperSignal West Pico chemiluminescent substrate (Thermo Scientific Pierce).

Identification of protein bands from SDS-PAGE was by mass spectrometry carried out at the Protein and Nucleic Acid Chemistry Facility of the University of Cambridge, Cambridge, United Kingdom. Samples were prepared according to the instructions given at http://www3.bioc.cam.ac.uk/pnac/proteomics.html.

### HT29 and Vero cell cytotoxicity assays

Overnight cultures of *C. difficile* in TY or TYG were used to inoculate media to a starting optical density at 600 nm of 0.05. Culture supernatants and bacterial cell lysates were collected at 6, 12, 18, and 24 h by centrifugation (4000 g, 10 min, 4 °C). Supernatants were filtered through 0.2 μm pore size units and concentrated on 3 kDa molecular weight cut-off centrifugal filters (Amicon). Bacteria were washed once in Tris-buffered saline (10 mM Tris.HCl, pH 7.5, 150 mM NaCl) and stored at −80 °C. Bacteria were re-suspended in PBS and lysed by repeated freeze-thawing at 37 °C. Lysates were centrifuged at 20, 800 g for 5 min and the supernatant removed into a fresh tube to obtain the soluble fraction. The preparations were stored at −20 °C. Samples were normalized by the addition of PBS to a final volume proportional to the optical density of the culture at the time of collection. For both soluble cell lysates and supernatants, an OD of 20 was diluted to a total volume of 50 μl in PBS. Ten-fold serial dilutions of these samples were made in PBS and 20 μl of each dilution was added to 80 μl of fresh media above confluent HT29 or Vero cells giving an additional 1-in-5 dilution. Cells were incubated (37 °C, 5 % CO_2_) with supernatants or lysates for 24 h before scoring toxin endpoint titers by examination using a Nikon Eclipse TS100 light microscope. Toxin end point-titers were taken as the first dilution at which cell morphology was indistinguishable from untreated controls. PBS alone or supernatants prepared from an isogenic *tcdA tcdB* [43] did not cause morphological changes in either cell line.

The HT-29 line is reported as being sensitive to TcdA in the pg range and TcdB in the mg range, whereas the Vero line is sensitive to TcdB in the pg range and TcdA in the ng range [32]. HT29 and Vero cells were cultured in Dulbecco’s modified Eagle’s medium (DMEM with 4500 mg/l glucose and sodium bicarbonate) supplemented with 10 % (v/v) fetal calf serum, 1 x non-essential amino acids, 4 mM L-glutamine and penicillin/streptomycin (Sigma). Confluent cell monolayers were prepared by seeding 96 well plates with 2 x 10^4^ cells/well followed by 72 h growth (37 °C, 5 % CO_2_).

### Availability of supporting data

Additional data supporting the results shown and the strains and plasmids generated in this study are available from the authors.

## Abbreviations

CDI: *C. difficile* infection
*mfd*: mutation frequency decline
CWP: Cell wall protein
PVDF: Polyvinylidene fluoride
CCR: Carbon catabolite repression
TCRF: Transcription-coupled repair factor
